# The Role of Copper in Bimetallic Nickel–Copper BEA Zeolite Catalysts and Their Activity in the Hydrocracking Process of Rapeseed Oil

**DOI:** 10.3390/ma19030518

**Published:** 2026-01-28

**Authors:** Łukasz Szkudlarek, Karolina Chałupka-Śpiewak, Aleksandra Zimon, Michał Jacek Binczarski, Waldemar Maniukiewicz, Paweł Mierczyński, Małgorzata Iwona Szynkowska-Jóźwik

**Affiliations:** Chemical Department, Institute of General and Ecological Chemistry, Lodz University of Technology, Zeromskiego 114, 90-543 Lodz, Poland; lukasz.szkudlarek@p.lodz.pl (Ł.S.); aleksandra.zimon@p.lodz.pl (A.Z.); michal.binczarski@p.lodz.pl (M.J.B.); waldemar.maniukiewicz@p.lodz.pl (W.M.); pawel.mierczynski@p.lodz.pl (P.M.); malgorzata.szynkowska@p.lodz.pl (M.I.S.-J.)

**Keywords:** bimetallic copper–nickel catalysts, BEA zeolite, hydrocracking of rapeseed oil

## Abstract

The main goal of this work was to determine the role of copper in bimetallic nickel–copper BEA zeolite catalysts prepared by sequential impregnation and co-impregnation, as well as defining the influence of the method of impregnation on the catalytic activity of the described catalysts. The all-prepared samples were tested in hydrocracking reactions with rapeseed oil as a feedstock. The physicochemical properties of the catalytic materials were determined using H_2_-TPR, TPD-NH_3_, XRD, BET, and SEM-EDS techniques. The reaction products were analyzed using chromatographic techniques (HPLC and GC-MS). The bimetallic systems obtained post-impregnation exhibited lower conversions of rapeseed oil than catalysts synthesized by co-impregnation. For all bimetallic catalysts, oil conversion was higher than for the monometallic copper BEA zeolite catalyst. This indicates that nickel is responsible for better oil conversion. The highest oil conversion (92.7%) was noted for the co-impregnated 5%Ni-5%Cu_BEA zeolite catalyst. For all tested catalysts, the highest selectivity was noted for the gasoil fraction. However, the presence of copper in bimetallic Cu-Ni_BEA zeolite catalysts led to increased selectivity towards gasoline and kerosene compared to the monometallic nickel BEA zeolite catalyst.

## 1. Introduction

The rapid development of the global economy with continuous improvements in industrialization has led to a continuous increase in energy demand, which is supplied largely through the use of fossil fuels (i.e., diesel, gasoline, kerosene, etc.). Unfortunately, such fuels are non-renewable, and, thus, the available supply is continually diminishing. The constantly growing consumption of conventional fuels results in increasing emissions of greenhouse gases (such as CO_x_, SO_x_, and NO_x_) and other pollutants that have a negative impact on the environment [1,2,3,4]. All of these factors have led us to search for alternative, renewable, environmentally friendly, and sustainable fuels, which can potentially substitute or replace conventional fuels [2].

One route for obtaining alternative fuels is the catalytic (hydro)cracking of vegetable oils, which can increase the activity and selectivity of liquid products and hydrocarbon compounds compared to thermal cracking [5]. The difference between these processes is the fact that catalytic cracking occurs without reacting with hydrogen gas, unlike hydrocracking [6]. The hydrocarbons produced in the hydrocracking process yield compounds with comparable properties to those found in conventional fuel. The alternative fuels from vegetable oils obtained by this route have higher energy densities, lower viscosity, higher storage stability, and a higher calorific value and cetane index [7,8]. Hydrocracking gives a wide range of hydrocarbon products, also known as green products, such as green naphtha (C_5_–C_9_), green gasoline (C_10_–C_12_), and green diesel (C_13_–C_20_) [3,9]. The use of vegetable oils as feedstock for hydrocracking fuels is advantageous because of the naturally occurring distribution of fatty acid chain lengths, where the reaction converts them to alkanes with boiling points near or above the high boiling point limit for commercial as well as military jet fuels [10]. An additional advantage of obtaining much more structurally complex products in hydrocracking is that they can be used not only in the energy sector as fuel, but also in the production of plastics, cosmetics, drugs, or surfactants [6].

This process requires a supported bifunctional metal/acid catalytic system. Crucial factors for the hydrocracking reaction are the physical properties of the support (e.g., pore size, and large BET surface area) and the balance between metallic and acidic sites on the catalyst [7,11]. The presence of acidic sites in the catalytic system allows for the cracking of molecules and provides isomerization, while metal sites are responsible for hydrogenation and dehydrogenation reactions [12]. Monometallic catalysts are commonly used for this process (the most common are the noble metals—Pt and Pd) [11], but not directly, due to the easy deactivation of the system, which translates into a reduction in catalytic activity, selectivity and resistance to coke deposition, along with the capability to sinter active metal species [5]. The second type of catalysts for the hydrocracking reaction are bimetallic catalytic systems based on non-noble metals, mainly derived from group VI (Mo and W) and group VIII (Ni and Co). The main advantage of the use of these systems is their lower cost in comparison to noble-metal-based catalysts [11]. Copper-based catalysts in hydroprocessing reactions have a preferential ability to hydrogenate C=O bonds present in carbonyl compounds, rather than hydrogenating C=C bonds [13,14]. The increase in active metallic sites in bimetallic catalysts is related to the dispersion of more metal ions on the surface of porous materials. This demonstrates the possibility of promoting catalytic activity along with selectivity and reducing the possibility of catalyst sintering during the reaction by immobilizing metal ions on supports characterized by large surface areas [15]. Typical supports with acidic properties for hydrocracking catalysts are amorphous oxides or mixtures of oxides (e.g., Al_2_O_3_, SiO_2_-Al_2_O_3_, and ZrO_2_/SO_4_^2−^), zeolites (e.g., zeolite Y, beta, mordenite, ZSM-5, and ZSM-22), or silicoaluminophosphates (such as SAPO-11, SAPO-31, and SAPO-41) [16]. Zeolites are readily used in hydrocracking reactions. These aluminosilicates are characterized by shape-selectivity, due to a well-defined pore system in their crystalline framework that allows them to promote or suppress specific reactions, which is related to the particle size of the reactants. In addition, they exhibit relatively strong acidity with a high surface area, and high thermal and hydrothermal stability. Furthermore, zeolites demonstrate enhanced resistance to poisoning as well as a reduced tendency to carbon deposition [17,18].

This work focuses on the catalytic activity of the BEA zeolite modified by nickel and copper as the active catalysts for the hydrocracking of rapeseed oil. In this work, the attempt was taken to explain the role of the order of the metal impregnation process on the catalytic activity of bimetallic Cu-Ni_BEA zeolite catalysts. The purpose of this study was also to determine the influence of the physicochemical properties of the prepared samples on their catalytic properties.

## 2. Materials and Methods

### 2.1. Materials Used for the Synthesis of Catalytic Systems

The ammonium form of BEA zeolite was purchased from Zeolyst International (Kansas City, KS, USA). The SiO_2_/Al_2_O_3_ molar ratio of the zeolite was 25. Nickel nitrate hexahydrate (Ni(NO_3_)_2_∙6H_2_O) (Sigma-Aldrich, St. Louis, MO, USA, purity 99.0%), and copper nitrate trihydrate (Cu(NO_3_)_2_∙3H_2_O) (CHEMPUR, Piekary Śląskie, Poland, purity 99.0%) were used as precursors of the active metal phases.

### 2.2. Preparation of Ni-Cu Catalysts

Prior to metal impregnation, the NH_4_BEA zeolite was calcined in a muffle furnace, in air at 500 °C for 20 h to obtain the protonated form (HBEA). Portions of HBEA zeolite were impregnated with aqueous solutions of nickel nitrate, copper nitrate, or a mixed solution containing both metal salts. In addition, sequential impregnation was performed by impregnating the Ni-loaded zeolite with a copper nitrate solution and Cu-loaded zeolite with a nickel nitrate solution. After impregnation, the samples were dried at 120 °C for 2 h and calcined at 550 °C for 4 h in air. The metal loading was 5 wt% for Ni and 5 wt% for Cu in both monometallic and also bimetallic catalysts.

### 2.3. Reaction Conditions

Hydrocracking of rapeseed oil was carried out in a 50 mL steel autoclave equipped with a mechanical stirrer (from Parr USA, Moline, IL, USA, reactor volume 50 mL). The reactions were conducted at 260 °C under hydrogen pressure of 50 bar for 2 h. Prior to catalytic testing, the calcined catalysts were reduced at 550 °C for 2 h in a 5%H_2_-95%Ar gas mixture at a flow rate of 50 cm^3^/min. All experiments were performed using catalyst amount of approximately 1 wt% relative to the oil mass. For the bimetallic 5%Ni-5%Cu_BEA zeolite catalyst prepared by co-impregnation, additional experiments were conducted at a reduced hydrogen pressure of 30 atm and a shorter time of 1 h to evaluate the influence of reaction condition on catalytic activity. Catalytic activity was expressed in terms of oil conversion and selectivity towards different products.

### 2.4. Analysis of Obtained Products

The analytical procedures applied for reagents and products were identical to those described in our previous work [19]. Oil conversion in hydrocracking reaction was determined by high-performance liquid chromatography (HPLC) using a SHIMADZU (Kyoto, Kyoto Prefecture, Japan) system equipped with a dual-piston pump (LC-10AT VP), autosampler (SIL-10AD VP HPLC), diode array detector (DAD, wavelength λ = 205 nm) (SPD-10A VP), low-pressure solvent mixer (FCV-10AL VP), and degasser (DGU-14A). An octadecyl C-18 column (5 µm, 4.6 × 250) was used. Reaction liquid samples were diluted with n-hexane at a ratio of 100 μL sample to 900 μL solvent. Methanol (Sovent A) and 2-propanol/hexane mixture (4:5 *v*/*v*, Solvent B) were used as the mobile phase. The applied gradient conditions are presented in Table 1.

Gas chromatography coupled with mass spectrometry (GC-MS) was used to determine the composition of vegetable oil hydrocracking reaction products. Sample preparation followed the same dilution procedure as for HPLC analysis. A Zebron, model ZB-5MSplus, capillary column was used, with length 30 m, inner diameter 0.25 mm, and layer thickness 0.25 μm. The detailed GC and MS operating conditions are summarized in Table 2, Table 3 and Table 4.

### 2.5. Catalysts Characterization

The specific surface area, pore volume, and average pore diameter of the catalysts were determined by the BET (Brunauer–Emmett–Teller) method based on low-temperature (77 K) nitrogen adsorption in ASAP 2020 apparatus (Micromeritics, MalvernPANalytical, Malvern, UK). Micropore volume and surface area were estimated using Dubinin–Radushkevich model and the t-plot method, while pore size distribution was determined using the BJH method.

Morphology, metal distribution, and elemental composition were analyzed by scanning electron microscopy coupled with energy-dispersive X-ray (SEM-EDS) performed on a S-4700 scanning electron microscope from HITACHI S-4700 equipped with EDS detector from Thermo Scientific (Waltham, MA, USA). Samples were placed on double-sided adhesive tape and fixed to SEM table, and then carbon-sputtered. The microscope was working at room temperature with an accelerating voltage of 25 kV and magnification of 1000–10,000× [19].

X-ray diffraction (XRD) measurements were carried out using a PANalytical X’Pert Pro MPD diffractometer (Malvern PANalytical, Malvern, UK). The X-ray source was a copper long fine focus X-ray diffraction tube operating at 40 kV and 30 mA. Data were collected in the 5–90° 2θ range within 0.0167° step. Crystalline phases were identified by references to the ICDD PDF-2 (version 2024) database. Data processing was performed with X’Pert HighScore Plus software ver. 4.9 (Malvern Panalytical Ltd., Malvern, UK) [19].

Temperature-programmed reduction (TPR) technique was used to determine the reducibility of the nickel–copper zeolite catalysts. The measurements were carried out on an automatic AMI-1 instrument (Altamira Instruments, Pittsburgh, PA, USA) equipped with a thermal conductivity detector. The reduction behaviors of the catalytic materials were measured in the temperature range of 35–900 °C with linear temperature increase of 10 °C/min and flow rate of reducing mixture of 5%H2-95%Ar equaling 40 mL/min [19].

Catalyst acidity was evaluated by ammonia temperature-programmed desorption (TPD). All measurements were carried out in a quartz-based home-made flow micro-reactor with the use of NH_3_ as a probe molecule. Prior to all experiments, the sample of catalyst was dried in flowing helium (flow rate: 40 cm^3^/min) at 550 °C for 1 h and reduced “in situ” at 550 °C for 2 h in reducing mixture (5%H_2_-95%Ar). The catalyst was then cooled down to 100 °C and ammonia was adsorbed on the surface of the catalyst for 15 min at 100 °C. After NH_3_ adsorption, the system was flushed with He for 15 min at 100 °C, in order to remove physiosorbed NH_3_ from the surface of catalyst. The sample was cooled to ambient temperature, and then the NH_3_-TPD experiment was carried out from 100 °C to 600 °C with a linear temperature increase of 25 °C/min. The desorption of NH_3_ gas from the acidic sites of the catalysts was identified via a thermal conductivity detector (TCD) [19].

## 3. Results

The catalytic performance of the studied catalyst samples can generally be related to their physicochemical properties. In this work, an attempt was made to explain the catalytic activity of bimetallic Ni-Cu_BEA zeolite catalysts by analyzing the role of copper and its influence on the physicochemical and, consequently, the catalytic behavior of the materials.

### 3.1. Catalytic Activity of Bimetallic Ni-Cu BEA Zeolite Catalysts

**Figure 1:**
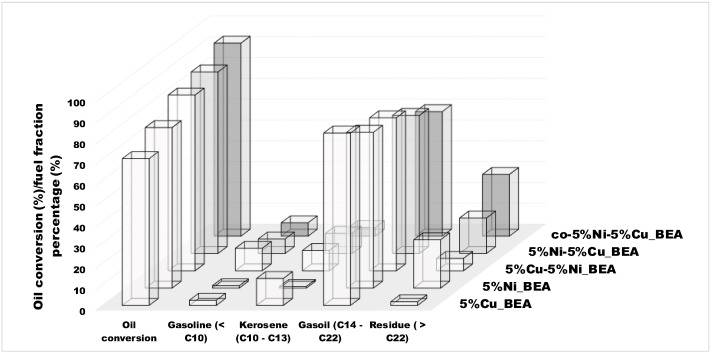
The oil conversion (%) and the selectivity to the fuel fraction.

**Figure 2:**
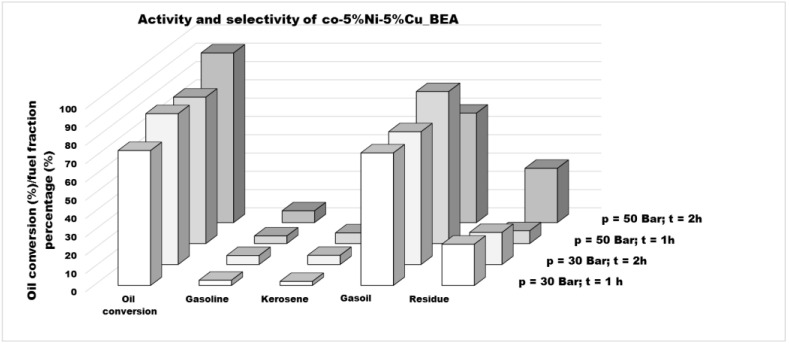
The comparison of activity and selectivity towards gasoline, kerosene, gasoil, and residue for co-5%Ni-5%Cu_BEA catalyst depending on reaction condition—hydrogen pressure and time of reaction.

The catalytic activity of each zeolitic catalysts in the reaction was expressed by the conversion of rapeseed oil and selectivity towards different hydrocarbons fraction divided into gasoline, kerosene, gasoil, and residue. The obtained results were collected in Table 5 and presented in Figure 1 and Figure 2.

The catalytic activity was considered as the degree of oil conversion. Both monometallic catalysts supported on BEA zeolite with a metal loading of 5% exhibited good catalytic activity in the hydrocracking process. The copper catalyst calcined and reduced at 300 °C achieved an oil conversion of 70.5%, whereas the nickel catalyst calcined and reduced at 550 °C under identical reaction conditions reached an oil conversion of 77.1%. In the case of bimetallic zeolite catalysts, it was found that the presence of both metals in BEA zeolite lead to an improvement in oil conversion. Moreover, the sequence of metal impregnation significantly influenced the catalytic performance. The 5%Cu-5%Ni/BEA system, prepared by a sequential impregnation with nickel followed by copper, gave a 84.4% conversion of triglycerides from rapeseed oil, in a hydrocracking reaction. In contrast, the reverse impregnation sequence (5%Ni-5%Cu/BEA) catalyst led to a slightly higher conversion of 87.2%. The best result was achieved in the reaction with the co-impregnated catalyst labelled as co-5%Ni-5%Cu/BEA, where the oil conversion was 92.7% (Table 5, Figure 1).

In this work, the influence of hydrogen pressure and reaction time on the catalytic activity of the co-impregnated bimetallic BEA zeolite catalyst was also investigated. Reducing the hydrogen pressure from 50 to 30 bar at a constant reaction time of 2 h resulted in a decrease in triglyceride conversion by aproximately 10%. Shortening the reaction time to 1 h at 50 bar led to an even more pronounced decrease of nearly 13%. When both the pressure and reaction time were reduced, the oil further declined to 73.7%, representing an overall decrease of approximately 20% (see Table 5 and Figure 2).

The second aspect which was considered as the measure of the application of mono- and bimetallic nickel–copper BEA zeolite catalysts for the hydrocracking process of vegetable oil was the selectivity towards various fraction of hydrocarbons. The product composition was determined by a GC-MS analysis, and the results are presented in Table 5 and Figure 1 and Figure 2. The following division into fuel fractions was adopted: the gasoline fraction for all hydrocarbons with the carbon number in the chain lower than 10; the kerosene fraction for hydrocarbons with the carbon number in the chain from 10 to 13; the gasoil fraction for hydrocarbons with carbon atoms from 14 to 22; and the residue fraction for all heavy hydrocarbons with the number of carbon atoms in the chain higher than 22. For all tested catalysts, the gasoil fraction constituted the dominant product (59.8–83.1%). In the case of monometallic catalysts supported on BEA zeolite, the copper catalyst exhibited selectivity towards athe gasoil fraction (82.8%), followed by the kerosene fraction (12.9%), with minor contributions from the gasoline (2.5%) and residue (1.8%) fractions. Interestingly, for the monometallic nickel catalyst, in addition to the significant content of the gasoil fraction (74.8%), hydrocarbons of the kerosene and gasoline fractions were found in very small amounts (0.8% and 1.1%, respectively), while hydrocarbons with a high number of carbon atoms in the molecule were much more noted. It could suggest that nickel favors the formation of hydrocarbons with longer chains. For bimetallic catalysts, the product selectivity was strongly influenced by the preparation method. For the reaction with a 5%Cu-5%Ni/BEA catalyst, where the zeolite was first impregnated with a nickel precursor solution and then impregnated with a copper precursor solution, the gasoil fraction (73.5%) contributed the most, of course, followed by gasoline and kerosene fractions with similar percentages (10.8% and 9.8%, respectively). On the other hand, for the 5%Ni-5%Cu/BEA system prepared by post-impregnation (first with a copper solution, then with a nickel solution) and the co-5%Ni-5%Cu/BEA catalyst prepared by co-impregnation, the reaction products presented lower contents of the gasoline (7.0% and 6.4%), kerosene (9.7% and 4.1%), and gasoil (66.3% and 59.8%, respectively) fractions, while a higher content of the residue fraction (17.1% and 29.7%) was reported. It is noteworthy that co-5%Ni-5%Cu/BEA exhibited the highest conversion of feedstock. For this catalyst, catalytic tests were also conducted for a reduced hydrogen pressure (from 50 bar to 30 bar) and reduced reaction time (from 2 h to 1 h). Reducing the reaction time while maintaining the pressure resulted in a higher proportion of the gasoil fraction (83.1%) in the products with little change in the percentages of the gasoline and kerosene fractions (4.2% and 5.8%, respectively), but with a significant reduction in the residue fraction (7.0%). In contrast, lowering only the pressure while maintaining the same reaction time yielded a similar content of hydrocarbons included in the gasoline and kerosene fractions (4.9% and 5.0%, respectively), while the content of the gasoil fraction (72.6%) and the residue fraction (17.5%) was increased. When both parameters were lowered, this reflected a very low content of the gasoline and kerosene fractions (2.8% and 2.2%, respectively) in the products while maintaining a similar percentage of hydrocarbons for the diesel phase (72.4%) and increasing the amount of residue (22.5%) compared to the reaction lasting 2 h at a hydrogen pressure of 30 bar. The general conclusion could be that the presence of copper in the bimetallic systems leads to an improvement in selectivity towards gasoline and kerosene, so it seems that copper is the factor which can be responsible for the formation of lighter hydrocarbons. Moreover, the presence of both metals in BEA zeolite catalysts generally caused an increase in selectivity toward gasoline and kerosene.

The similar trend was reported by Nur Aini et al. [20] for the Ni-Cu/HZSM-5 catalyst used in the hydrocracking of Cerbera manghas oil. The authors achieved the best oil conversion (48.59%) and yield to biofuel products (47.86%) in the reaction with a 5% loading of Ni-Cu/HZSM-5 (metal ratio—1:2) under the temperature of 375 °C for 2 h. In addition, the authors also achieved the highest gasoil selectivity (c. 90%) with little selectivity towards kerosene. The hydrocracking products contained between 12 and 23 carbon atoms. In another work [21], Ni-Cu/HZSM-5 catalysts with a metal ratio of 1:1 have been studied. The process was carried out at 375 ˚C under an initial pressure of 11 bar for 2 h. The main products of hydrocracking were C_14_-C_20_ hydrocarbons. The yield towards diesel was 82.7% for the Ni-Cu/HZSM-5 system (1:1) at a catalyst loading of 5%, while, at a catalyst loading of 10%, the diesel yield was 23.75%.

### 3.2. The Specific Surface and Morphology of Mono- and Bimetallic Copper–Nickel BEA Zeolite Catalysts

The textural properties of the studied catalytic systems were determined using the Brunauer–Emmett–Teller (BET) method and the results were presented in Table 6. The obtained results pointed to small changes in the specific surface area (SSA) and micropore surface area (MSA) and a decrease in these values (about 23–40 m^2^/g) when both metals, Cu and Ni, are present in the catalytic systems. The biggest decline in the specific surface area and micropore surface area was found for the 5%Cu-5%Ni_BEA zeolite catalyst. The smallest decline in SSA and MSA was noted for the bimetallic co-5%Ni-5%Cu_BEA prepared by co-impregnation and it can suggest that the distribution of metal particles was more homogenous than in the case of samples prepared by sequential impregnation. Changes in the micropore volume and average pore size were not noted. For all investigated samples, these values were similar. Moreover, we did not observe a significant decline in the value of the external surface area (ESA), which can indicate that metal ions are present in various positions in the BEA zeolite framework, and they are probably not cumulated on the external surface, but, rather, well-distributed. In relation to the catalytic activity results, it seems that the textural properties of copper–nickel BEA zeolite catalysts do not affect in a significant way the level of vegetable oil conversion and selectivity towards different hydrocarbons.

**Figure 3:**
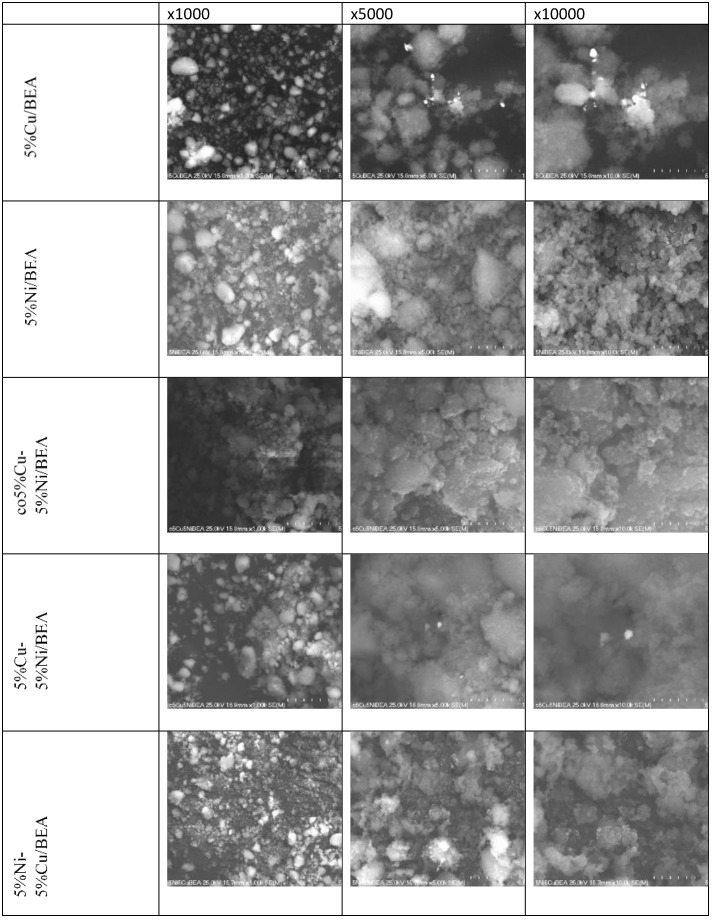
SEM images of catalysts after calcination at 550 °C for 4 h at magnification of ×1000, ×5000, and ×10,000.

The SEM analysis (Figure 3) revealed slight morphological differences depending on the catalyst preparation method. For the monometallic 5%Cu_BEA and bimetallic 5%Cu–5%Ni_BEA catalyst prepared by sequential impregnation, small and well-dispersed metallic agglomerates, typically below 0.5 µm, were observed on the surface.

The EDS analysis presents the elemental composition consistent with the designed catalyst formulation as shown in Figure S1 in the Supplementary Material. These spectra confirmed the presence of both Cu and Ni species on the BEA zeolite surface, indicating the successful incorporation of the metallic components.

### 3.3. The Phase Composition and Reducibility of Bimetallic Cu-Ni BEA Zeolite Catalysts

**Figure 4:**
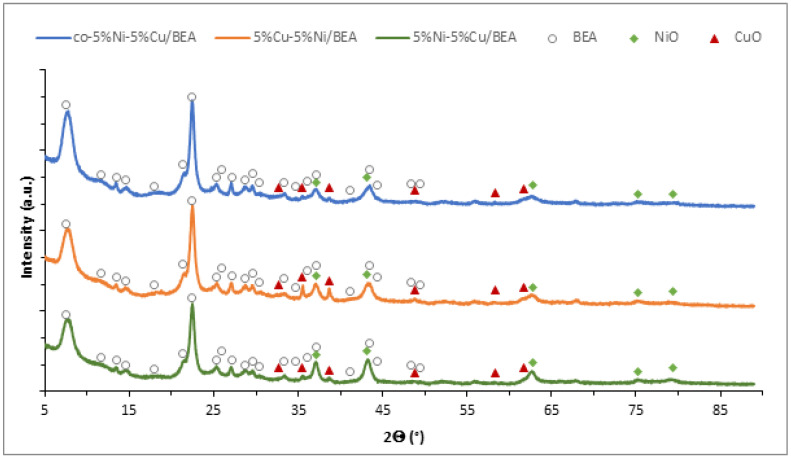
XRD patterns for bimetallic nickel–copper BEA zeolite after calcination at 550 °C for 4 h.

With the purpose of determining the phase composition of the obtained catalytic materials for the hydrocracking reaction, X-ray diffraction studies were carried out. Diffractograms of the investigated nickel–copper zeolite catalysts are illustrated in Figure 4. The XRD patterns exhibit characteristic diffraction peaks originating from BEA zeolite, nickel oxide, and copper oxide. Reflections assigned to the NiO phase are observed at 2θ angle values of 37.2°; 43.2°; 62.8°; 75.3°; and 79.4°. In the diffractograms, diffraction peaks for the CuO phase were observed at the 2θ angles = 32.7°; 35.5°; 38.7°; 48.8°; 58.3°; and 61.7°. In the study of Ni–Cu bimetallic zeolites catalysts performed by Zheng and co-authors [22], diffraction peaks originating from nickel oxide were seen on the XRD pattern at 2θ = 37.5° and 43.5°. The authors also confirmed that reflections at 2θ = 38.7° and 48.6° are the characteristic diffraction peaks of CuO. Dahou et al. [23] observed characteristic diffraction peaks at the 2θ angles of 32.64°, 35.71°, 39.09°, 48.91°, 53.58°, 58.31°, 61.64°, 66.48°, and 68.22°, which are attributed to the (110), (002), (111), (202), (020), (202), (113), (311), and (220) crystal planes. In the case of the NiO crystallographic phase, Aboul-Elein et al. [24] observed diffraction peaks at 2θ angle values of 37.2°, 43.2°, 62.8°, 75.4°, and 79.4°, which are assigned to the (111), (200), (220), (311), and (222) planes. This was also confirmed in the work by Derikvandi and co-researchers [25], where XRD lines were observed at 2θ = 37.26°, 43.3°, 69.89°, 75.43°, and 79.43°, respectively. The individual reflections are attributed to the following crystal planes (111), (200), (220), (311), and (222), respectively. The other reflections observed on the diffractograms at 2θ angle values of 7.5°; 11.6°; 13.5°; 14.6°; 17.9°; 21.4°; 22.4°; 25.3°; 25.9°; 27.1°; 28.7°; 29.6°; 30.4°; 33.3°; 34.7°; 36.1°; 37.1°; 41.2°; 43.5°; 44.3°; 48.4°; and 49.5° are characteristic of BEA zeolite. The general conclusion is that the BEA zeolite structure was preserved after impregnation. Moreover, in the case of bimetallic copper–nickel BEA zeolite samples, the presented results indicated the presence of both nickel and copper oxides on the external surface and in the bulk of zeolite.

**Figure 5:**
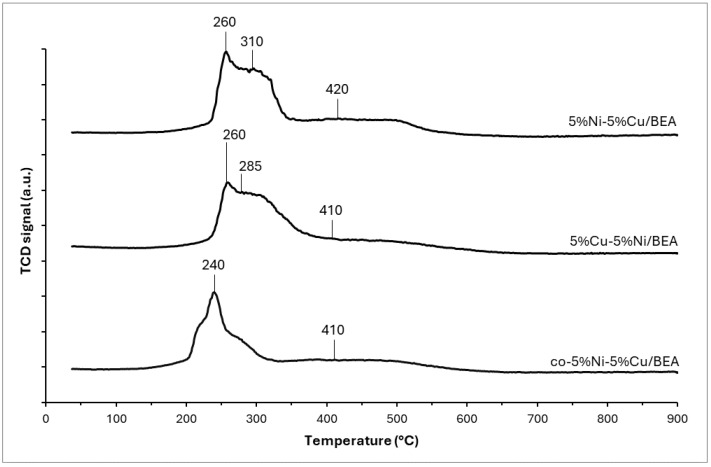
TPR profiles for bimetallic copper–nickel BEA zeolite catalysts after calcination at 550 °C for 4 h.

With the aim of determining the reducibility of the obtained modified BEA zeolite catalysts with nickel and copper, TPR-H_2_ measurements were carried out. The resulting profiles are presented in Figure 5. The analysis of the TPR profile of the bimetallic 5%Ni-5%Cu/BEA catalytic system showed three reduction peaks with the maximum at 240–260 °C, 285–310 °C, and 410–420 °C.

The first and second reduction effects visible on the profiles of all bimetallic catalysts originate from the two-stage sequential reduction of copper (II) oxide (Cu^2+^ → Cu^+^ → Cu^0^). Lower values of the maximum temperature of these peaks in the case of the coimpregnated co-5%Ni-5%Cu/BEA zeolite catalyst compared to the other studied samples are associated with particle dispersion. In this co-5%Ni-5%Cu/BEA system, the dispersion is probably greater; thus, there are CuO particles, which are more easily reducible than bulk CuO particles, because of the relatively larger surface area to react with hydrogen [26]. Such a low temperature of the maximum of the first reduction peak suggests that there is no interaction between CuO particles and zeolite, or this interaction is weak. This may also indicate that the CuO particles are present on the surface of the zeolite rather than inside the pores of the zeolite [27].

The last peak in the profiles of bimetallic Ni-Cu zeolite systems is related to the reduction of bulk nickel oxide, which usually occurs at 400–450 °C [28]. According to Yin and co-authors [29], at this temperature, the reduction of bulk nickel species with a weak interaction with the support occurs. It should be noted, however, that the latter peak is broad and its tail ends behind the temperature of 600 °C, indicating that Ni species that exhibit a stronger interaction with the zeolite are present. Typically, the reduction temperature of pure NiO is around 420 °C; however, it is known that the addition of CuO in the catalytic system can reduce it, due to the spillover effect [30]. This phenomenon involves the dissociation of H_2_ molecules into hydrogen atoms, which occurs on metal particles—in this case, metallic copper particles. As a result, the created hydrogen atoms have great activity which helps in the reduction of nickel species in the studied systems [31]. The confirmation of the decrease in the reduction temperature of nickel oxide in the catalyst by the addition of CuO might be provided by referring the obtained results for bimetallic Ni-Cu zeolite systems to the work written by Hamidzadeh et al. [32], where, for a sample of the monometallic Ni(N)/ZSM-5 catalyst synthesized using a nitrate precursor, a peak corresponding to bulk NiO reduction with a maximum of 473 °C and a second peak at 648 °C were observed on the TPR profile, indicating the presence of strong nickel-carrier interactions and well-distributed Ni particles inside the zeolite.

**Figure 6:**
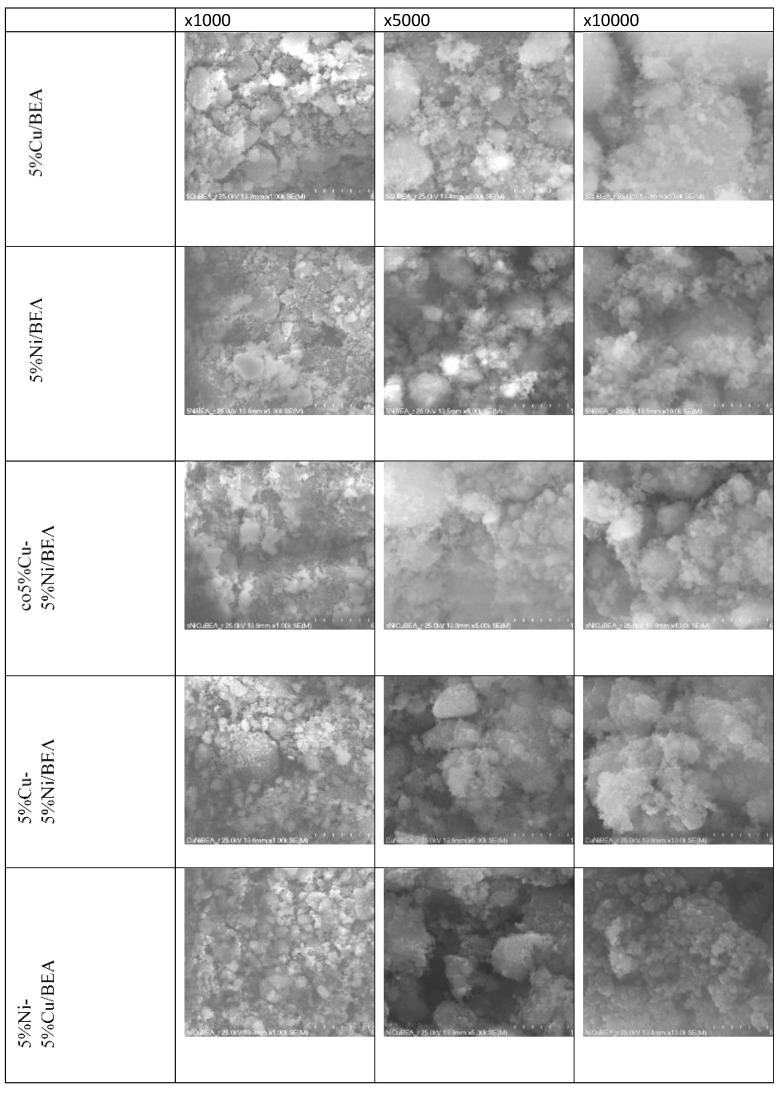
SEM images of catalysts after reduction at magnifications of ×1000, ×5000, and ×10,000.

With the aim of checking the morphology after the reduction process, the SEM-EDS analysis was carried out. The obtained results were presented in Figure 6 and in Figure S2 in the Supplementary Material. After reduction, the catalysts exhibited a more homogeneous and compact surface morphology compared to the calcined samples. The SEM images showed that the metallic particles are well-dispersed over the BEA zeolite surface, and no distinct metal agglomerates are observed. This indicates that the reduction process promotes a uniform distribution of the active phase and contributes to the stabilization of the catalyst structure. These observations suggest that the reduction step improves the dispersion of metallic species and enhances the structural integrity of the catalysts, regardless of the metal deposition sequence or preparation route.

### 3.4. The Acidity of Mono- and Bimetallic Copper–Nickel BEA Zeolite Catalysts

The acidity of monometallic and bimetallic copper and nickel BEA zeolite catalysts was measured using the temperature-programmed desorption of ammonia. The obtained results for the samples after calcination and reduction were collected in Table 7. In the case of bimetallic copper–nickel BEA zeolite catalysts, it was observed that the presence of nickel led to a decrease in total acidity in comparison to the monometallic 5%Cu_BEA zeolite sample. A similar observation was noted by Li and co-authors [33], whose TPD experiments studied both copper and nickel catalytic systems supported on ZSM-5 zeolite. The authors observed that the total acidity was slightly weaker for the nickel catalyst than for the copper zeolite system. They also observed on the TPD profile two peaks, which were assigned to the weak and strong acid sites on the surfaces. In their work was found, for the copper catalyst, a broad peak from 150 °C to 400 °C, with a maximum at 297 °C assigned to weak acid centers, and a second peak from 400 °C to 600 °C with a maximum at 459 °C originating from strong acid centers. In contrast, for the nickel catalyst, the maxima of these peaks were 216 °C and 376 °C, respectively.

In the case of bimetallic copper–nickel BEA zeolite catalysts, the total acidity was the same as noted for the monometallic 5%Ni_BEA zeolite sample and it was equal to near 2.5 mmol/g. The smallest total acidity equaling 2.37 mmol/g was observed for the bimetallic co-5%Ni-5%Cu_BEA zeolite catalyst prepared by the co-impregnation method. For this sample, the smallest external surface area was also observed, which can suggest that the preparation of this catalytic system by the co-impregnation method causes the accumulation of metal oxides in the pore of zeolite and the same blocking access to acidic sites in BEA zeolite. The highest decline in the number of acidic centers was observed for the moderate acid site for all studied bimetallic catalysts. However, the number of these acidic sites for all bimetallic samples was almost the same and it equaled 0.90–0.92 mmol/g. It should also be mentioned that the amount of strong acidic sites was the smallest for all investigated catalysts. A higher difference in the values of the acidic sites was noted for the weak acidic sites and the smallest amount was found in the case of the co-5%Ni-5%Cu_BEA zeolite catalyst. This can suggest that the decrease in acidity can improve the conversion of vegetable oil.

Gutta and coworkers [35], in their paper, observed a similar dependancy. They observed, for all studied bimetallic Cu-Ni catalysts, desorption peaks below 200 °C, attributed to the presence of a large number of weak acid sites, and, for systems containing aluminum in addition to silicon in the support (MCM-41), the profiles showed additional desorption peaks in the temperature ranges of 250–275 °C and 350–480 °C corresponding to moderate and strong acid sites. The occurrence of differences in the acidity of the studied systems were explained by the authors through the occurrence of different interactions between Ni and Cu with the present Al species (mainly Al^3+^) in the zeolitic framework and the order of impregnation, as a result of which there is a different content of Ni and Cu on the surface and inside the framework of the zeolite systems prepared by the post-impregnation and co-impregnation methods. All this translates into a different content (in a change) in the number of Lewis and/or Brønsted acid sites in the obtained Ni-Cu catalytic systems on BEA zeolite.

## 4. Discussion

Attention should be given to the processes related to the transformation of vegetable oils into bio-fuel, including determining the economics, availability, and production yield. The vegetables oils represent an attractive renewable feedstock for biofuel production due to their availability, liquid state, and ease of processing. [36]. The obtained results suggests that activation by the reduction process is a good way for the preparation of active catalysts for the hydrocracking process and this process improves the dispersion of metal species, the active center in the BEA zeolite matrix. The XRD and SEM-EDS investigations point to the gaining of very well-dispersed metal ions in the BEA zeolite matrix which can be responsible for the better reducibility of active centers in catalytic systems and, thus, for better activity in hydrocracking process. The well-dispersed metal species and their presence in various positions in the BEA zeolite framework can lead to a decrease in the acidity of the BEA zeolite. The catalytic activity results indicate that the copper addition and also the bigger accessibility of this metal on the external surface of BEA zeolite in the sample of 5%Cu-5%Ni_BEA, prepared by the post-impregnation method, led to an increase in selectivity towards gasoline and kerosene—the desired products in bio-jet fuel. The same dependency in selectivity towards different fuel fractions was observed for the monometallic 5%Cu_BEA zeolite catalyst. In the case of the monometallic 5%Ni_BEA zeolite catalyst, the increase in oil conversion was observed, but, simultaneously, the highest selectivity towards the residue fraction containing the hydrocarbons with a long carbon chain (C > 22) was found. For all bimetallic copper–nickel BEA zeolite catalysts, the rise in oil conversion and also the selectivity towards the harder hydrocarbon fraction was noted. Hydrocracking favors the C-C bond breakage and reduces the molecular weight giving the gasoline-range fuel. The hydrocracking of vegetable oils usually leads to the production of hydrocarbons with carbon in the chain from C_12_ to C_18_ [36]. Our studies pointed out that even a small addition of copper can cause an increase in lighter hydrocarbon production. Simultaneously, the decline in selectivity towards the gasoline, kerosene, and gasoil fraction was discovered in comparison to the monometallic 5%Cu_BEA zeolite catalyst. This can suggest that nickel can be responsible for the better oil conversion, but it also caused an increase in selectivity towards harder hydrocarbons. Similar observations were made by Q. Guo and co-workers, who concluded that the addition of copper to bimetallic Ni-Cu/ZrO_2_ led to a decrease in the reduction temperature of the active sites and an increase in the resistance to carbon deposition, and, finally, an improvement in the efficiency of this catalyst in the hydrodeoxygenation of algae bio-oil [37]. However, the mechanism is complicated because, during the hydrocracking of vegetable oils, different reactions can occur. Four different mechanisms for hydrocracking reactions can be proposed, including the bifunctional mechanism, hydrogenolysis, thermal cracking, and the Haag–Dessau mechanism [36].

It seems that the combination of both metals in BEA zeolite catalysts lead to an improvement in oil conversion, and, in future studies, various combinations of copper to nickel in different ratios can allow for the modification and control of the kind of products formed. It is also very likely that a modification in the metal ratio can cause differences in selectivity towards various fuel fraction. In the case of the co-impregnated co-5%Ni-5%Cu_BEA zeolite sample, it was observed that the decrease in reaction time and, also, the decline in hydrogen pressure caused a reduction in oil conversion and increase in selectivity towards gasoil.

## Figures and Tables

**Table 1:** Phase gradient used in the HPLC measurements [19].

Mobile Phase Gradient			Flow Rate [mL/min]
Time [min]	Solvent A (%)	Solvent B (%)	
0	100	0	0.9
20	100	0	0.9
45	0	100	0.9
70	0	100	0.9
75	100	0	0.9

Solvent A: Methanol; Solvent B: 2-Propanol/Hexane = 4/5; Injection Volume: 1 μL; Column Temperature: 25 °C.

**Table 2:** Analysis settings of gas chromatography apparatus GC-2010 [19].

Column Oven Temperature	35.0 °C
Injection Temperature	320.00 °C
Injection Mode	Split
Injection Volume	1.00 μL
Flow Control Mode	Linear Velocity
Pressure	22.9 kPa
Total Flow	10.7 mL/min
Column Flow	0.70 mL/min
Linear Velocity	30.0 cm/s
Purge Flow	3.0 mL/min
Split Ratio	10.0

**Table 3:** Settings for oven temperature program [19].

Rate (°C/min)	Temperature (°C)	Hold Time (min)
-	35.0	5.00
15.00	320.0	6.00

**Table 4:** Analysis settings of mass spectrometer (GCMS-QP2010 SE, Shimadzu Corporation, Kyoto, Japan) coupled to gas chromatography apparatus GC-2010 [19].

Ion Source Temperature	220.00 °C
Interface Temperature	280.00 °C
Solvent Cut Time	2.50 min
Detector Gain Mode	Relative to the Tuning Result
Detector Gain	0.74 kV + 0.00 kV
Threshold	0
Start Time	2.70 min
End Time	30.00 min
ACQ Mode	Scan
Event Time	0.30 s
Scan Speed	1666
Start *m*/*z*	35.00
End *m*/*z*	500.00
Sample Inlet Unit	GC

**Table 5:** The comparison of catalytic activity and selectivity of mono- and bimetallic Cu-Ni_BEA zeolite catalysts in the hydrocracking of rapeseed oil depending on the reaction conditions.

Catalyst	Reaction Conditions H_2_ Pressure (Barr)/Time (h)	Oil Conversion (%)	Selectivity (%)			
			Gasoline (<C10)	Kerosene (C10–C13)	Gasoil (C14–C22)	Residue (>C22)
5%Cu_BEA	50 Barr/2 h	70.5	2.5	12.9	82.8	1.8
5%Ni_BEA		77.1	1.1	0.8	74.8	23.2
5%Cu-5%Ni_BEA		84.4	10.8	9.8	73.5	6.0
5%Ni-5%Cu_BEA		87.2	7.0	9.7	66.3	17.1
co-5%Ni-5%Cu_BEA	50 Barr/2 h	92.7	6.4	4.1	59.8	29.7
	50 Barr/1 h	80.0	4.2	5.8	83.1	7.0
	30 Barr/2 h	82.4	4.9	5.0	72.6	17.5
	30 Barr/1 h	73.7	2.8	2.2	72.4	22.5

**Table 6:** The textural properties of mono- and bimetallic copper–nickel BEA zeolite catalysts: specific surface area, micropore surface area, external surface area, micropore volume, and average pore size.

Catalyst	SSA (m^2^/g)	MSA (m^2^/g)	ESA (m^2^/g)	Micropore Volume (cm^3^/g)	Average Pore Size (nm)
5%Cu_BEA	489.8	306.1	183.6	0.16	14.4
5%Ni_BEA	496.9	307.1	189.8	0.16	14.4
5%Cu-5%Ni_BEA	454.9	268.6	186.3	0.14	14.5
5%Ni-5%Cu_BEA	462.4	276.5	186.0	0.14	15.0
co-5%Ni-5%Cu_BEA	473.9	293.3	175.7	0.15	13.8

**Table 7:** The acidity expressed as amount of ammonia adsorbed calculated from the TPD-NH_3_ data.

Catalyst	Total Acidity (mmol/g) 100–600 °C	Distribution of Acid Sites			Reference
		Weak (mmol/g) 100–300 °C	Moderate (mmol/g) 300–500 °C	Strong (mmol/g) 500–600 °C	
5%Cu_BEA calcined	3.33	1.54	1.03	0.76	[34]
5%Cu_BEA reduced at 300 °C 2 h	3.05	1.02	1.24	0.79	
5%Ni_BEA reduced at 500 °C 1 h	2.53	-	-	-	This work
5%Cu-5%Ni_BEA reduced at 500 °C 1 h	2.53	0.88	0.92	0.73	This work
5%Ni-5%Cu_BEA reduced at 500 °C 1 h	2.55	0.98	0.90	0.66	
co-5%Ni-5%Cu_BEA reduced at 500 °C 1 h	2.37	0.74	0.91	0.72	

## Data Availability

The original contributions presented in this study are included in the article/Supplementary Materials. Further inquiries can be directed to the corresponding author.

## Supplementary Materials

The following supporting information can be downloaded at: https://www.mdpi.com/article/10.3390/ma19030518/s1. Figure S1. The X-ray characteristic radiation spectra for (a) 5%Cu/BEA, (b) 5%Ni/BEA, (c) co5%Cu-5%Ni/BEA, (d) 5%Cu-5%Ni/BEA, (e) 5%Ni-5%Cu/BEA catalysts after calcination. Figure S2. The X-ray characteristic radiation spectra for (a) 5%Cu/BEA, (b) 5%Ni/BEA, (c) co5%Cu-5%Ni/BEA, (d) 5%Cu-5%Ni/BEA, (e) 5%Ni-5%Cu/BEA catalysts after reduction.
