# Supplementary material for: The Role of Copper in Bimetallic Nickel–Copper BEA Zeolite Catalysts and Their Activity in the Hydrocracking Process of Rapeseed Oil
Source: Materials (Basel). 2026 Jan 28;19(3):518. doi: 10.3390/ma19030518 (PMC12897874; doi:10.3390/ma19030518)
Supplement: Supplementary file 1 [file materials-19-00518-s001.zip › materials-4061103-supplementary.pdf]

Supplementary material to the manuscript:

The role of copper in bimetallic Nickel-Copper BEA zeolite catalysts for their activity in the hydrocracking process of rapeseed oil

Łukasz Szkudlarek, Karolina Chałupka-Śpiewak*, Aleksandra Zimon, Michał Binczarski, Waldemar Maniukiewicz, Paweł Mierczyński, Małgorzata Iwona Szynkowska-Jóźwik

[1] Lodz University of Technology, Chemical Department, Institute of General and Ecological Chemistry, Zeromskiego 114, 90 – 543 Lodz

* Correspondence: karolina.chalupka@p.lodz.pl; Tel.: +48 0-42 631 30 93

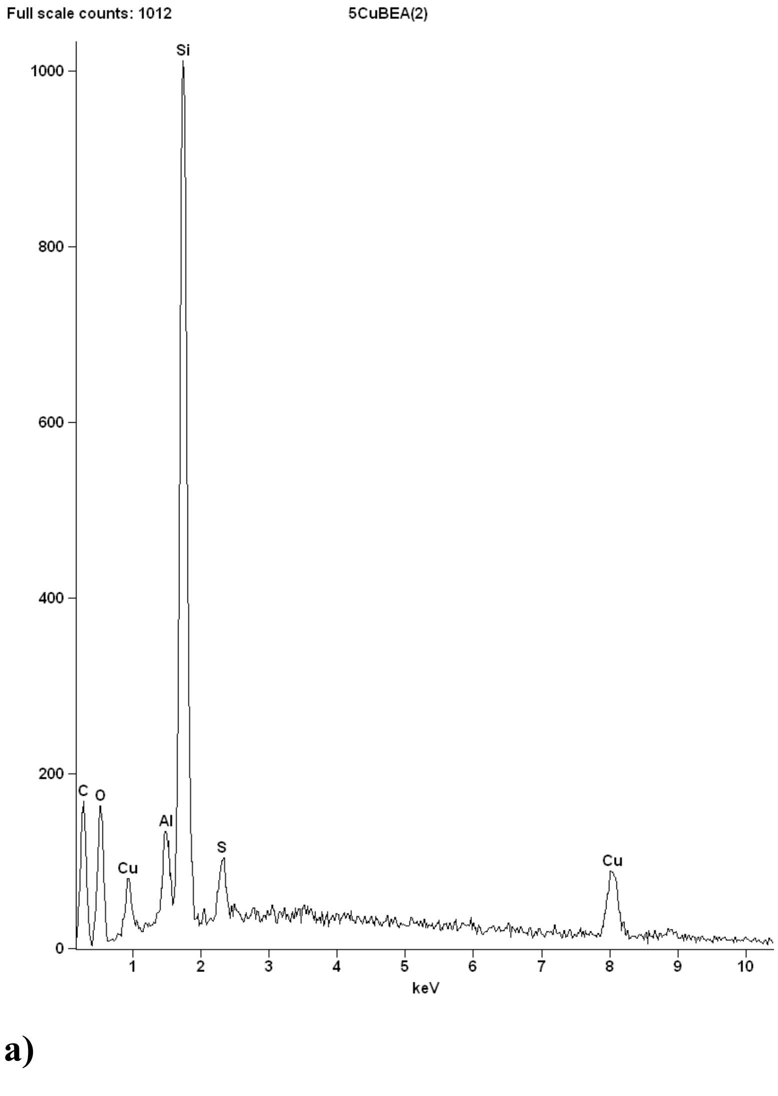

a)

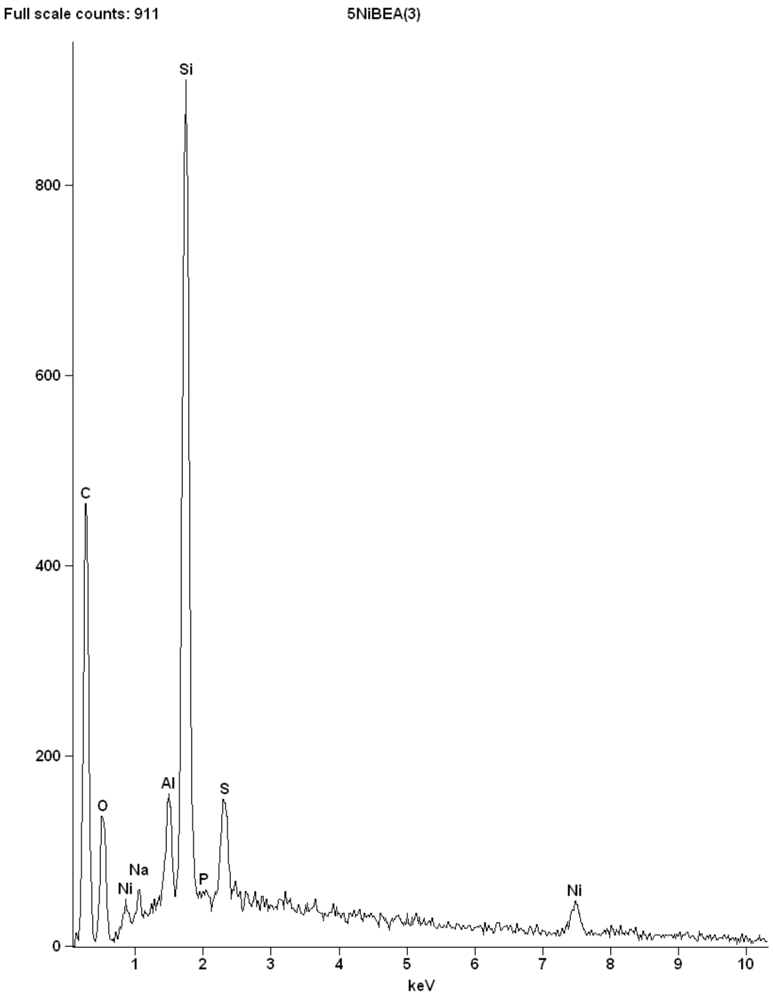
Full scale counts: 911
5NiBEA(3)
Si
C
O
Al
S
Na
Ni
P
Ni
800
600
400
200
0
1
2
3
4
5
6
7
8
9
10
keV

b)

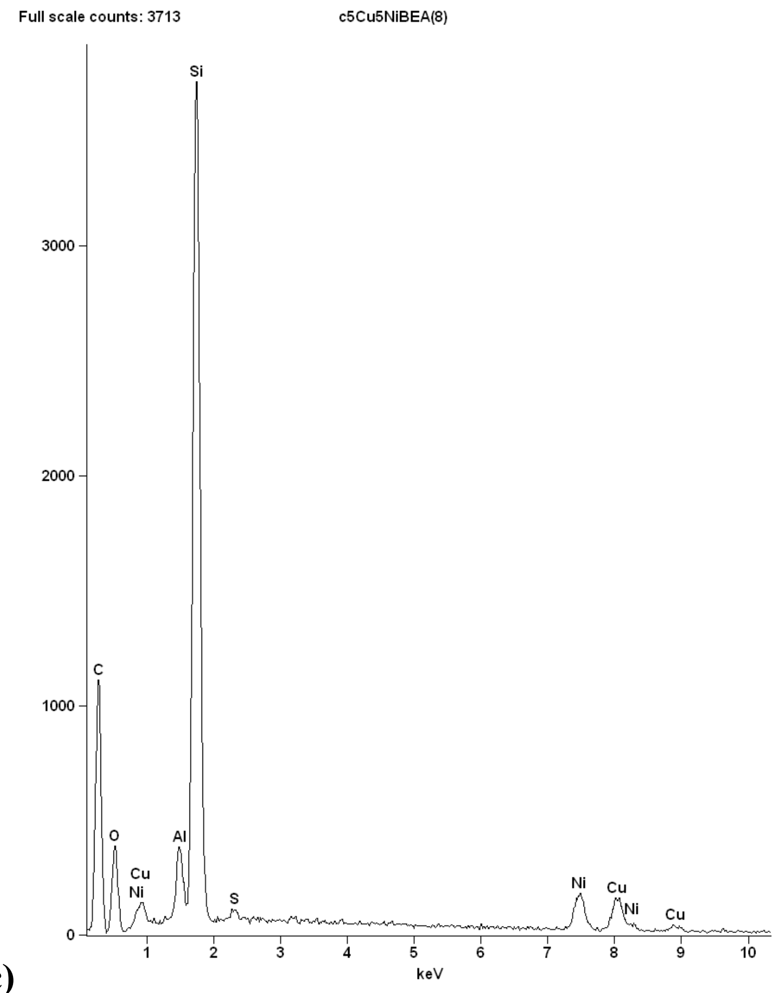
Full scale counts: 3713
c5Cu5NiBEA(8)
Si
3000
2000
C
1000
O
Al
Cu
Ni
S
Ni
Cu
Ni
Cu
0
1
2
3
4
5
6
7
8
9
10
keV

c)

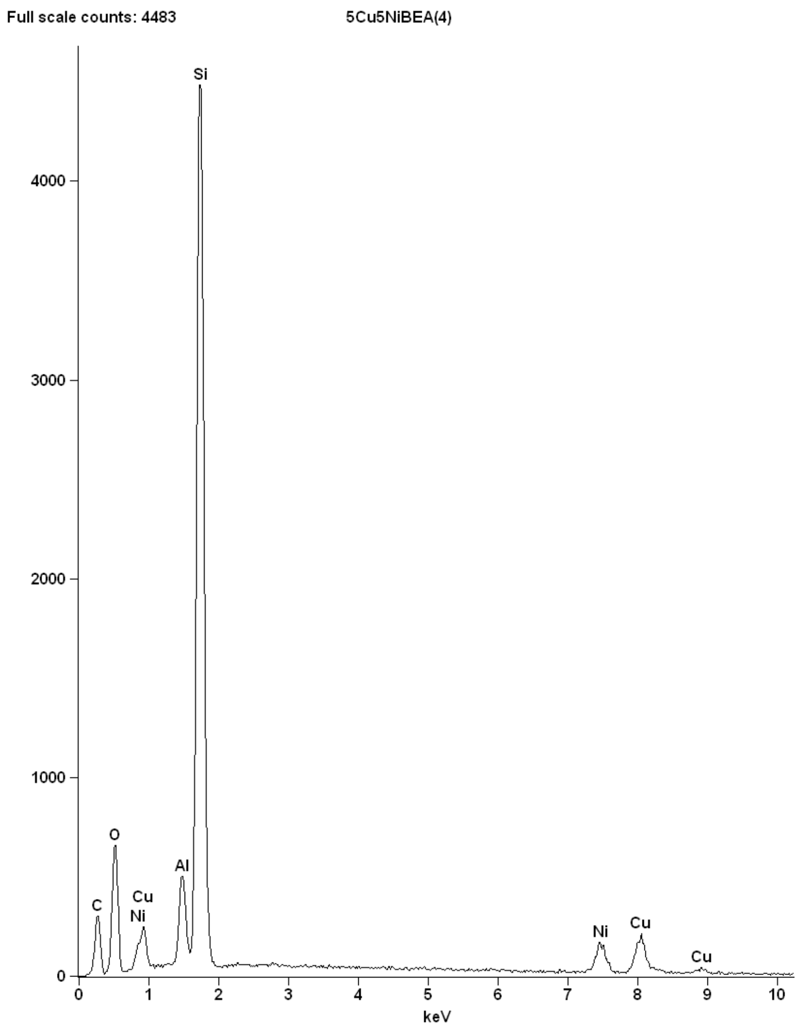
Full scale counts: 4483
5Cu5NiBEA(4)
Si
4000
3000
2000
1000
0
O
Al
C
Cu
Ni
Ni
Cu
Cu
0 1 2 3 4 5 6 7 8 9 10
keV

d)

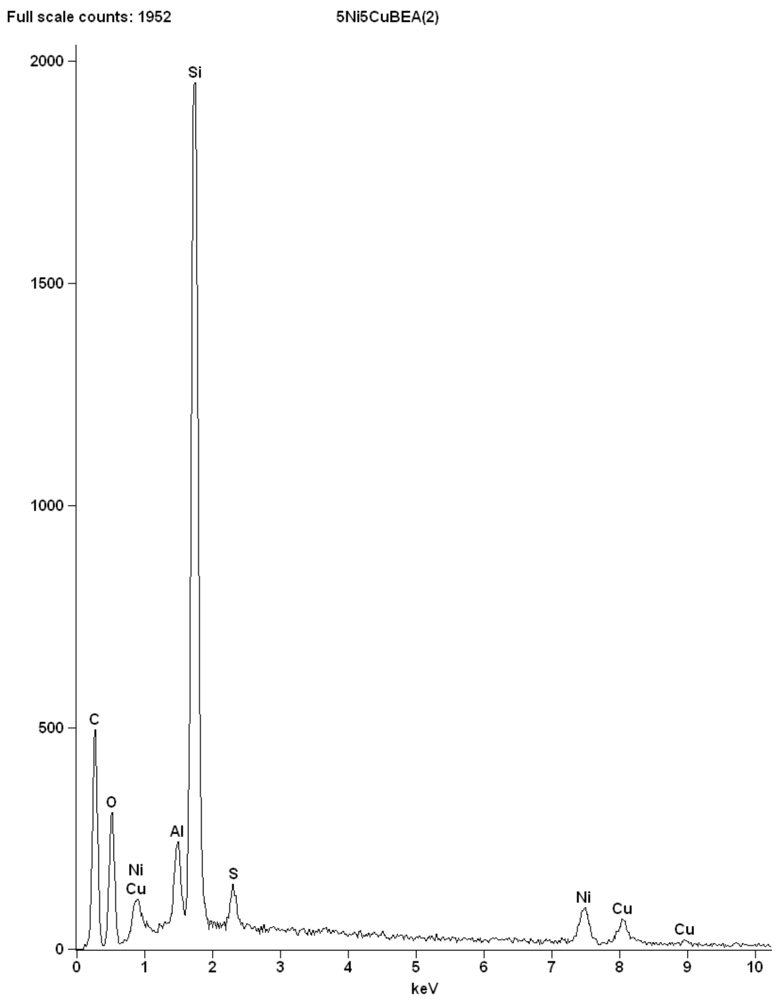

e)

Figure S1. The X-ray characteristic radiation spectra for a) 5%Cu/BEA, b) 5%Ni/BEA, c) co5%Cu-5%Ni/BEA, d) 5%Cu-5%Ni/BEA, e) 5%Ni-5%Cu/BEA catalysts after calcination.

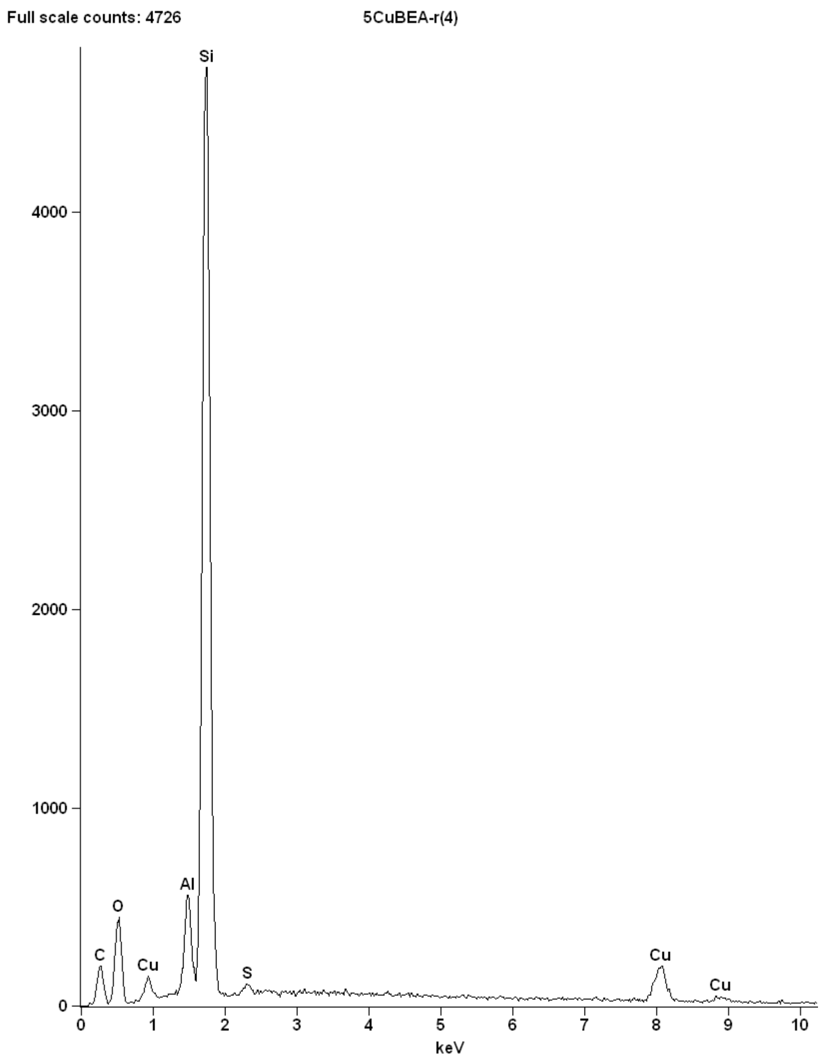

Full scale counts: 4726
5CuBEA-r(4)
Si
4000
3000
2000
1000
0
Al
O
C
Cu
S
Cu
Cu
0
1
2
3
4
5
6
7
8
9
10
keV

a)

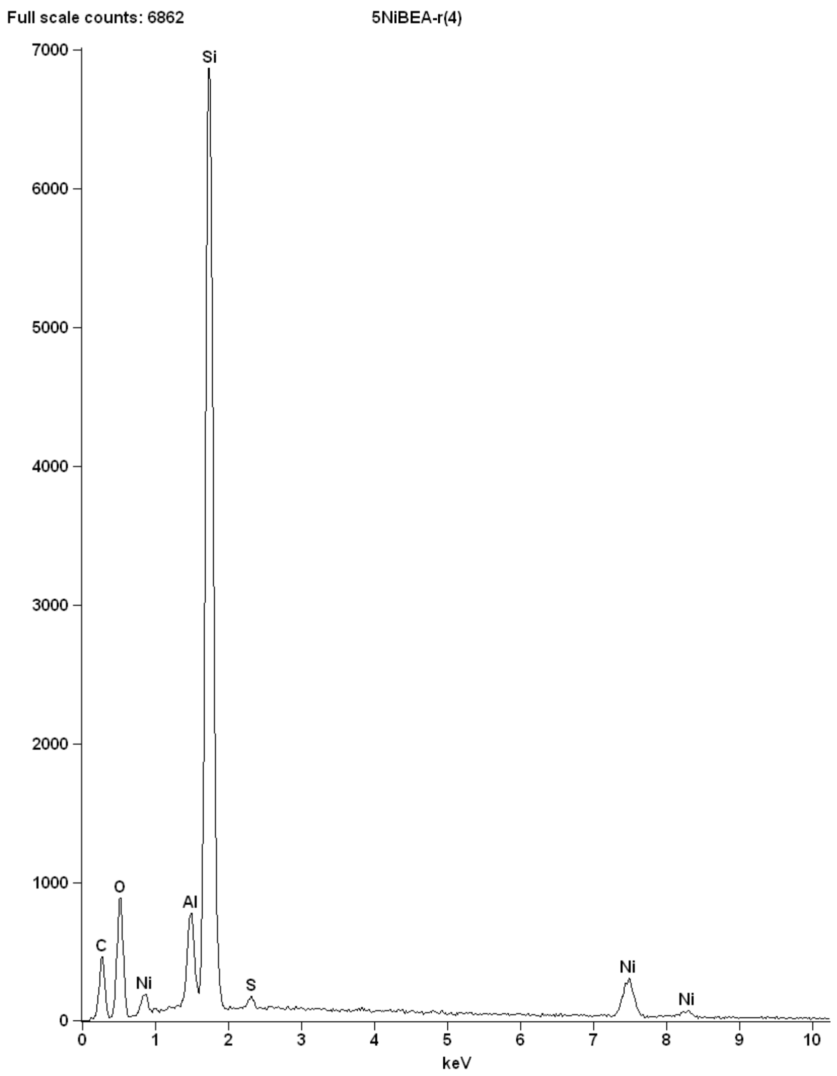
Full scale counts: 6862
5NiBEA-r(4)
7000
6000
5000
4000
3000
2000
1000
0
Si
O
Al
C
Ni
S
Ni
Ni
0
1
2
3
4
5
6
7
8
9
10
keV

b)

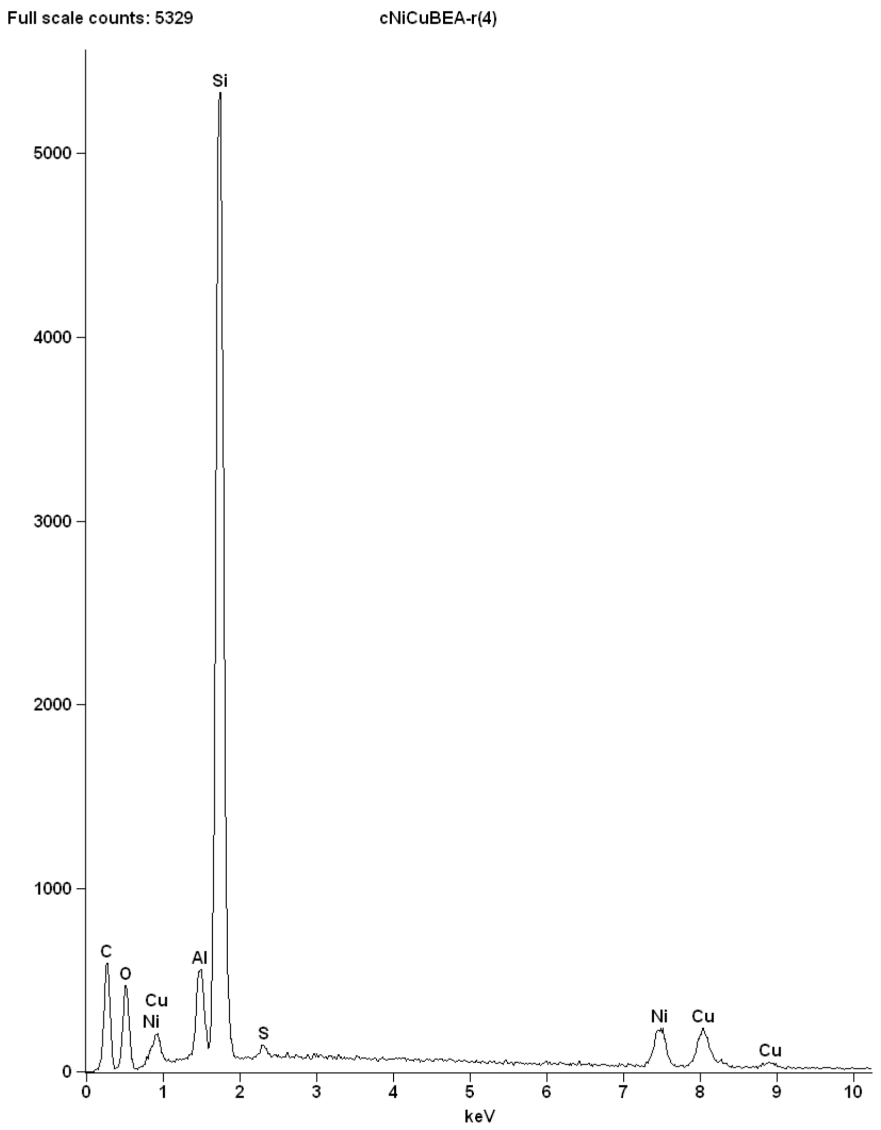
Full scale counts: 5329
cNiCuBEA-r(4)
Si
5000
4000
3000
2000
1000
0
C
O
Cu
Ni
Al
S
Ni
Cu
Cu
0
1
2
3
4
5
6
7
8
9
10
keV

c)

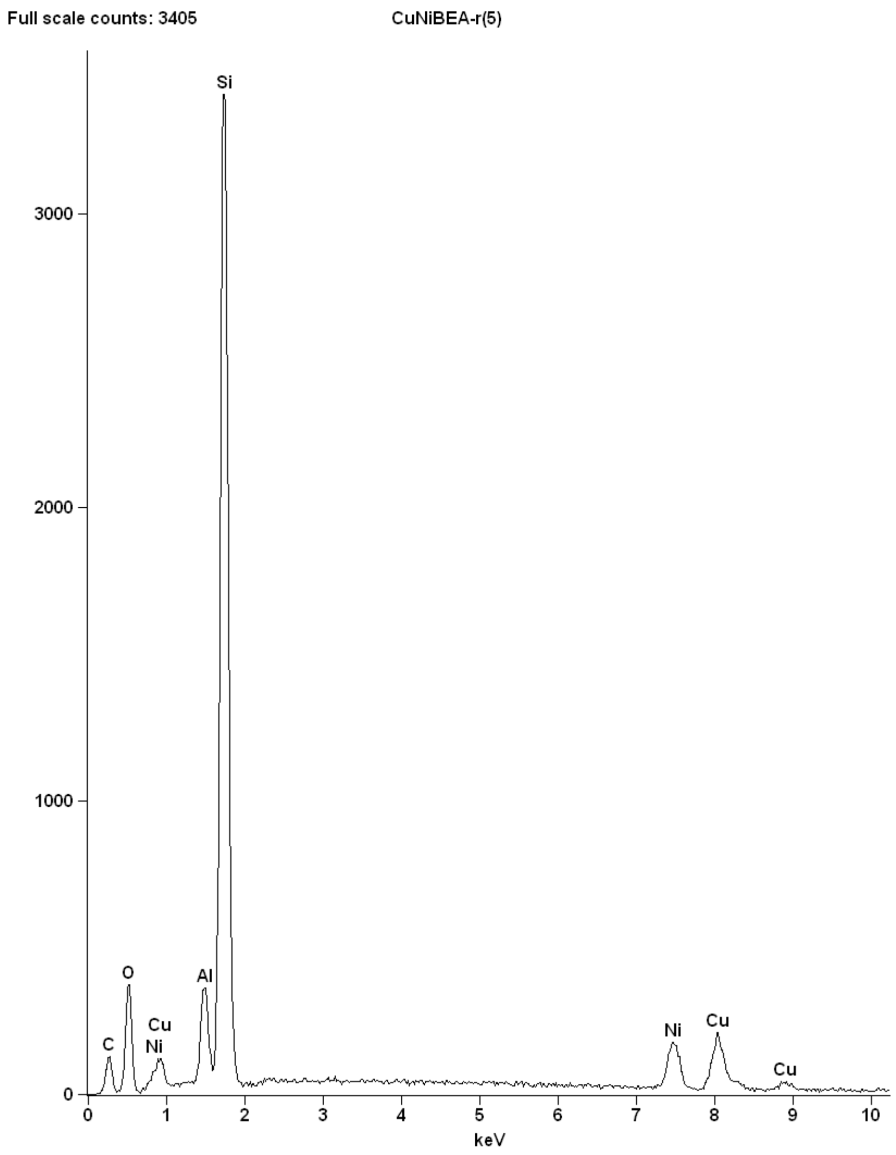
Full scale counts: 3405
CuNiBEA-r(5)
Si
3000
2000
1000
0
O
Al
Cu
Ni
C
Ni
Cu
Cu
0
1
2
3
4
5
6
7
8
9
10
keV

d)

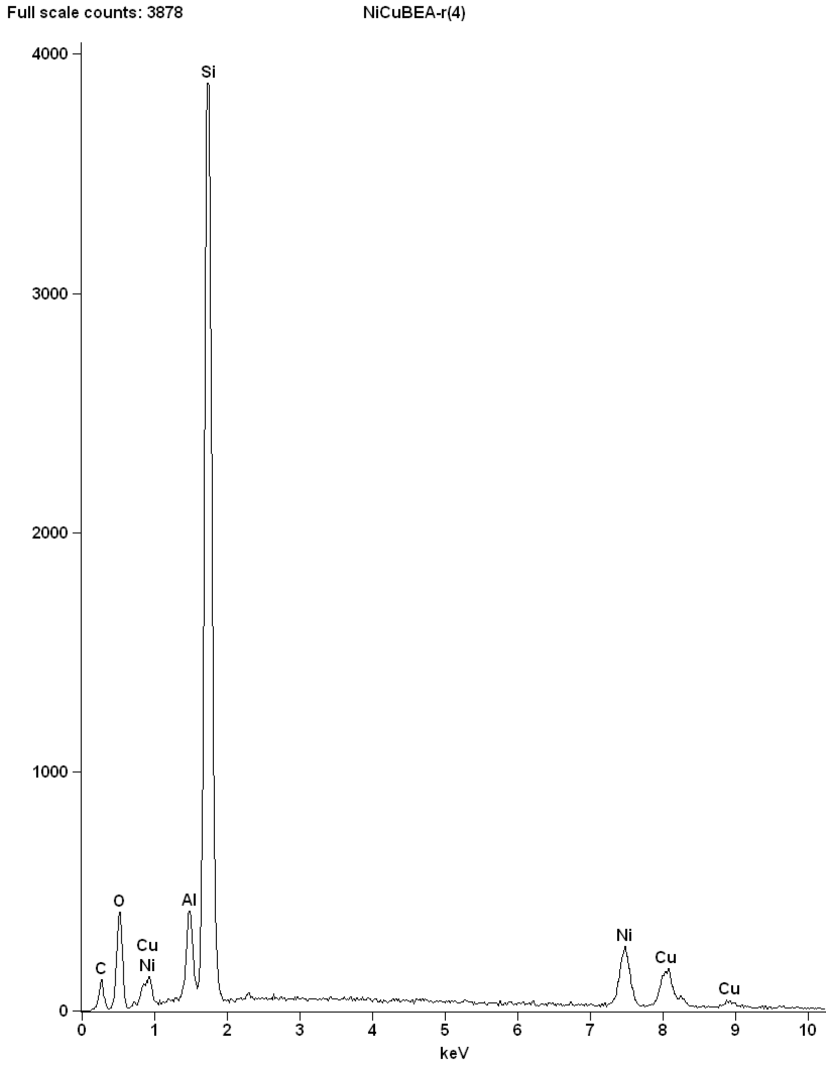

e)

Figure S2. The X-ray characteristic radiation spectra for a) 5%Cu/BEA, b) 5%Ni/BEA, c) co5%Cu-5%Ni/BEA, d) 5%Cu-5%Ni/BEA, e) 5%Ni-5%Cu/BEA catalysts after reduction.
